# Asprosin Exerts Pro-Inflammatory Effects in THP-1 Macrophages Mediated via the Toll-like Receptor 4 (TLR4) Pathway

**DOI:** 10.3390/ijms24010227

**Published:** 2022-12-23

**Authors:** Kiran Shabir, Seley Gharanei, Sophie Orton, Vanlata Patel, Parbata Chauhan, Emmanouil Karteris, Harpal S. Randeva, James E. Brown, Ioannis Kyrou

**Affiliations:** 1 Warwick Medical School, University of Warwick, Coventry CV4 7AL, UK; 2 Warwickshire Institute for the Study of Diabetes, Endocrinology and Metabolism (WISDEM), University Hospitals Coventry and Warwickshire NHS Trust, Coventry CV2 2DX, UK; 3 Aston Medical School, College of Health and Life Sciences, Aston University, Birmingham B4 7ET, UK; 4 College of Health, Medicine and Life Sciences, Brunel University London, Uxbridge UB8 3PH, UK; 5 Centre for Sport, Exercise and Life Sciences, Research Institute for Health & Wellbeing, Coventry University, Coventry CV1 5FB, UK; 6 Laboratory of Dietetics and Quality of Life, Department of Food Science and Human Nutrition, School of Food and Nutritional Sciences, Agricultural University of Athens, 11855 Athens, Greece

**Keywords:** asprosin, adipokines, inflammation, THP-1 macrophages, Toll-like receptor 4, TLR4

## Abstract

Adipose tissue is a dynamic endocrine organ, secreting a plethora of adipokines which play a key role in regulating metabolic homeostasis and other physiological processes. An altered adipokine secretion profile from adipose tissue depots has been associated with obesity and related cardio-metabolic diseases. Asprosin is a recently described adipokine that is released in response to fasting and can elicit orexigenic and glucogenic effects. Circulating asprosin levels are elevated in a number of cardio-metabolic diseases, including obesity and type 2 diabetes. In vitro studies have reported pro-inflammatory effects of asprosin in a variety of tissues. The present study aimed to further elucidate the role of asprosin in inflammation by exploring its potential effect(s) in THP-1 macrophages. THP-1 monocytes were differentiated to macrophages by 48 h treatment with dihydroxyvitamin D3. Macrophages were treated with 100 nM recombinant human asprosin, 100 ng/mL lipopolysaccharide (LPS), and 10 μM caffeic acid phenethyl ester (CAPE; an inhibitor of NFκB activation) or 1 µM TAK-242 (a Toll-like receptor 4, TLR4, inhibitor). The expression and secretion of pertinent pro-inflammatory mediators were measured by qPCR, Western blot, ELISA and Bioplex. Asprosin stimulation significantly upregulated the expression and secretion of the pro-inflammatory cytokines: tumour necrosis factor α (TNFα), interleukin-1β (IL-1β), IL-8 and IL-12 in vitro. This pro-inflammatory response in THP-1 macrophages was partly attenuated by the treatments with CAPE and was significantly inhibited by TAK-242 treatment. Asprosin-induced inflammation is significantly counteracted by TLR4 inhibition in THP-1 macrophages, suggesting that asprosin exerts its pro-inflammatory effects, at least in part, via the TLR4 signalling pathway.

## 1. Introduction

Obesity prevalence has nearly tripled worldwide since the 1970s [1], and is now recognized as a major contributor to the development of a number of cardio-metabolic disorders, including type 2 diabetes mellitus (T2DM) and cardiovascular disease (CVD) [2,3]. Excess energy storage as fat in adipocytes is the hallmark of obesity, with adipocytes undergoing cellular hypertrophy and hyperplasia under conditions of a chronic positive energy balance [4]. Accumulation of large adipocytes within white adipose tissue overtime in obesity can progressively result in a hypoxic local environment and tissue fibrosis, ultimately leading to reduced adipose tissue plasticity, dysfunction and cell death [5]. Dysfunctional and apoptotic adipocytes are shown to release pro-inflammatory mediators that promote the infiltration of immune cells into the adipose tissue and initiate a local immune response. Hence, obesity, depending on its degree and duration, is typically associated with the development of a chronic, low-grade, inflammatory state [6,7]. Immune cells (e.g., macrophages) are now recognized as a key component of adipose tissue, involved in removing apoptotic cells [8], by forming crown-like structures around apoptotic cells and remove cellular debris [9]. In obesity, resident macrophages are the most abundant immune cell type that accumulate within adipose tissue depots [10]. Of note, macrophages primarily express two main phenotypes, namely M1 (classical) and M2 (alternative), which promote and suppress inflammation, respectively. The switch between these phenotypes is regulated by the signals received from other immune cells and adipocytes [11,12]. In lean adipose tissue, interleukin-13 (IL-13) and IL-14, released by eosinophils and T-helper-2 (Th2) cells polarise macrophages to a M2 phenotype to promote an anti-inflammatory environment [13,14]. However, in obese adipose tissue, pro-inflammatory signals secreted by other activated immune cells (e.g., dendritic cells, mast cells, Th1 cells, neutrophils, CD8+ T-cells) and dysfunctional adipocytes can polarise macrophages to the M1 phenotype [10]. Moreover, the M1 macrophages release pro-inflammatory mediators, such as tumour necrosis factor alpha (TNFα), IL-6 and IL-8, which further exacerbate adipose tissue inflammation [6,15].

Overall, adipose tissue is now regarded as a dynamic endocrine organ, which in obesity exhibits a significantly altered secretory profile of multiple adipose-derived, pleiotropic factors, collectively called adipokines [16]. Asprosin is a glucogenic and orexigenic adipokine, which is released by white adipose tissue in response to fasting [17,18]. This novel adipokine is encoded in the last two exons (65 and 66) of the FBN1 gene and is the c-terminal cleavage product of a larger pro-protein, i.e., pro-fibrillin [17]. Thus, asprosin is 140-amino acids long (approximately 30 kDa), with three glycosylation sites in two of the amino acid sequences [17,19]. Romere et al. (2016) first discovered that asprosin is released from white adipose tissue during a fasting state and acts on the liver to induce glucose secretion via the G-protein-cAMP-protein kinase A (PKA) pathway [17]. Later, Li et al. (2019) found that asprosin acts through the G-protein coupled receptor OLFR734 in the liver; the human ortholog of this receptor is considered to be the olfactory receptor 4M1 (OR4M1) [20,21]. Notably, asprosin can also cross the blood–brain barrier and has been shown to act on appetite-regulating, agouti-related protein (AgRP+) neurons in the brain to promote satiety and reduce energy expenditure via the G-protein-cAMP-PKA pathway [18]. Accordingly, Olfr734 knockdown in mice has been shown to reduce appetite and stunt the activation of AgRP+ neurons following administration of asprosin [22].

Moreover, circulating asprosin levels are reported to be elevated in a certain cardio-metabolic diseases, including obesity, T2DM, gestational diabetes mellitus and polycystic ovary syndrome [23]. In addition, both in vitro and in vivo studies have reported pro-inflammatory effects of asprosin on skeletal muscle cells and pancreatic beta-cells, resulting in reduced insulin signalling and secretion, respectively [24,25]. Additional in vivo experiments revealed that asprosin augmented glucose intolerance, insulin resistance, endoplasmic reticulum (ER) stress, as well as circulation of pro-inflammatory cytokines (monocyte chemoattractant protein-1, MCP-1; IL-6; and TNFα) in mice [24]. Asprosin appears to mediate these effects via the protein kinase C delta (PKCδ)-sarco/endoplasmic reticulum Ca2+-ATPase 2 (SERCA2) pathway, since silencing of PKCδ reversed these asprosin-induced effects [24]. Furthermore, given the detrimental effects of free fatty acids on pancreatic beta cells [26,27,28], Lee et al. (2019) also found that asprosin mediates pro-inflammatory effects of palmitate, a saturated fatty acid, on pancreatic beta cells. In this study, palmitate-treated beta cells were shown to release asprosin, which acted in an autocrine manner to induce inflammation, apoptosis and ER stress, consequently impairing insulin secretion by these cells [25]. Silencing of Toll-like receptor 4 (TLR4) and c-Jun N-terminal kinase (JNK) suppressed these asprosin-mediated increase in pro-inflammatory cytokine expression and cell death, whilst improved cell viability and insulin secretion [25]. Thus, these findings suggest that the TLR4-JNK pathway is responsible for the effects of asprosin on pancreatic beta-cell dysfunction.

Collectively, these data indicate that asprosin may exert pleiotropic actions, contributing to the metabolic and pro-inflammatory dysfunction observed in obesity-related cardio-metabolic diseases. To date, there is a paucity of data on the potential effects of asprosin on macrophage function, despite the well-established role of macrophages as key regulators of the pro-inflammatory state in obesity. As such, the aim of the present study was to investigate the potential effects of this novel adipokine on THP-1 macrophages.

## 2. Results

### 2.1. Asprosin Increases the Expression and Release of Pro-Inflammatory Molecules from THP-1 Macrophages

**Figure 1:**
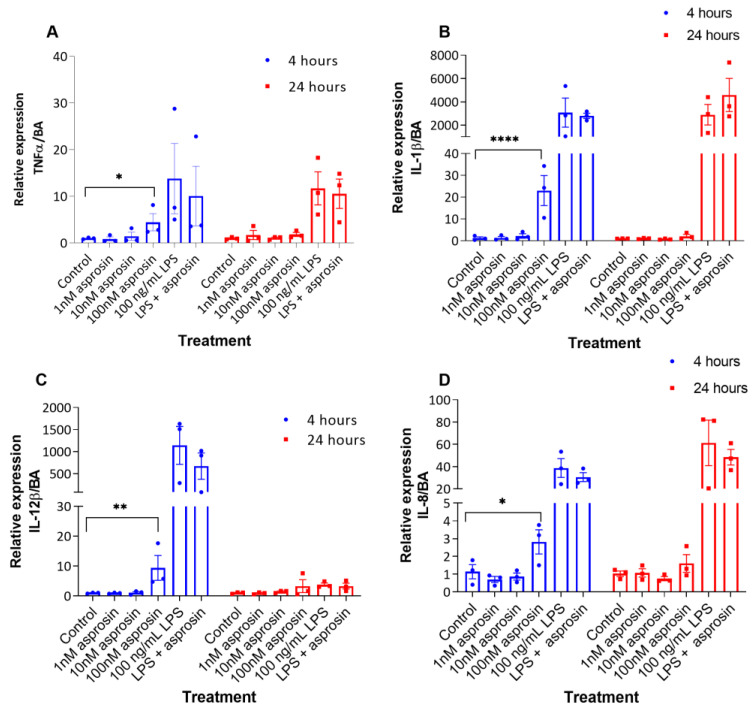
Gene expression of pro-inflammatory cytokines increased after 4 h of treatment with 100 nM asprosin. THP-1 macrophages were treated with increasing concentrations of asprosin (1 nM, 10 nM, and 100 nM), 100 ng/mL lipopolysaccharide (LPS) or both 100 ng/mL and 100 nM asprosin for 4 h and 24 h. (**A**) Tumour necrosis factor α (TNFα); (**B**) Interleukin-1β (IL-1β); (**C**) IL-12β; and (**D**) IL-8 mRNA quantification by RT-qPCR. Values were compared using two-way ANOVA and Tukey’s multiple comparisons test (compared to respective controls). Data are presented as mean ± SEM; n = 3; * *p* < 0.05, ** *p* < 0.01; **** *p* < 0.0001.

**Figure 2:**
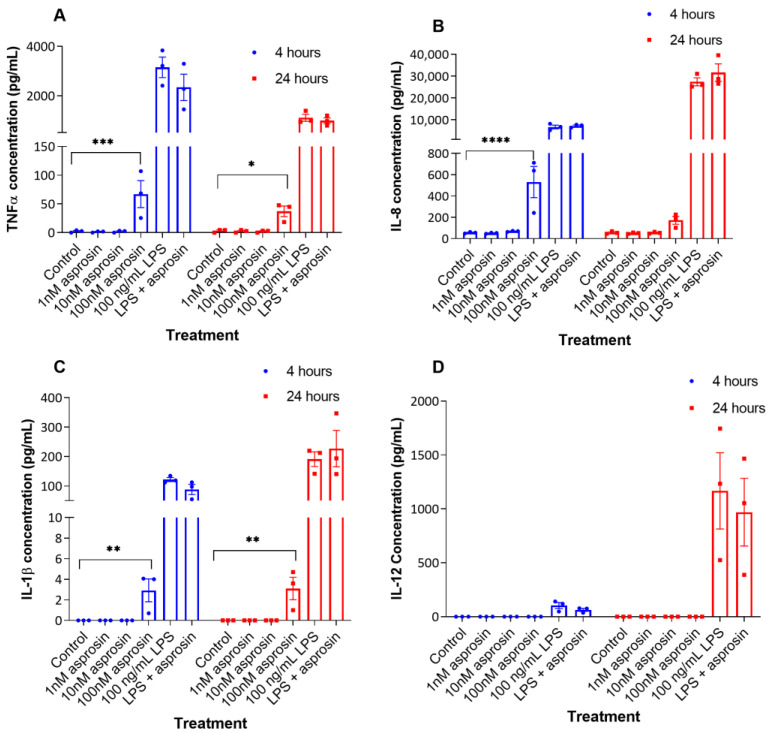
Asprosin treatment (100 nM) increases the release of tumour necrosis factor α (TNFα), interleukin-8 (IL-8) and IL-1β by THP-1 macrophages. THP-1 macrophages were treated with increasing concentrations of asprosin (1 nM, 10 nM, and 100 nM), 100 ng/mL lipopolysaccharide (LPS) or both 100 ng/mL and 100 nM asprosin for 4 h and 24 h. Cell supernatants were collected and (**A**) TNFα, (**B**) IL-8, (**C**) IL-1β, and (**D**) IL-12β concentrations were measured by ELISA. Data were analysed by two-way ANOVA and Tukey’s multiple comparisons test (compared to respective controls). Data are presented as mean ± SEM; n = 3; * *p* < 0.05; ** *p* < 0.01; *** *p* < 0.001; **** *p* < 0.0001.

The pro-inflammatory effects of asprosin have been shown in skeletal muscle cells and pancreatic beta-cells [24,25], we therefore sought to investigate the effects of asprosin on gene expression and release of key pro-inflammatory cytokines in THP-1 macrophages. Based on the previous studies [24,25], we used a concentration range of 1 nM, 10 nM and 100 nM asprosin for 4 h and 24 h (Figure 1 and Figure 2). Since a 100 nM treatment showed an effect at 4 h, this concentration and time course was used for the rest of the experiments. Compared to unstimulated control, qPCR data revealed that treatment with 100 nM asprosin significantly increased the gene expression of TNFα (*p* = 0.026), IL-1β (*p* < 0.0001), IL-12β (*p* = 0.007) and IL-8 (*p* = 0.012) after 4 h (Figure 1). No significant difference in gene expression between asprosin-treated cells and control was noted after 24 h of this treatment. Lipopolysaccharide (LPS 100 ng/mL, as used by previous studies in macrophages [29,30]) was used as a positive control. Unlike in asprosin-treated cells, gene expression levels of all cytokines, except IL-12β, remained high after 24 h of LPS exposure (Figure 1).

Secretory levels of these pro-inflammatory cytokines in response to asprosin were also measured by ELISA. There were no differences in the levels of these pro-inflammatory cytokines in the cell culture supernatants of THP-1 macrophages treated with lower asprosin concentrations (1 nM and 10 nM) (Figure 2). However, compared to untreated cells, 100 nM asprosin significantly increased the release of TNFα into the surrounding cell media after 4 h (2.4 ± 1.3 pg/mL vs. 67.1 ± 23.59 pg/mL, respectively; *p* = 0.0003), but not after 24 h (Figure 2A). Compared to control, IL-8 levels were also significantly higher in the cell culture supernatant after a 4 h treatment with 100 nM asprosin (55.45 ± 3.87 pg/mL vs. 530.12 ± 145.92 pg/mL, respectively; *p* < 0.0001) (Figure 2B). Moreover, 100 nM asprosin stimulated the release of IL-1β after 4 h (2.93 ± 1.11 pg/mL; *p* = 0.0047), and 24 h (3.10 ± 1.11 pg/mL; *p* = 0.0029) (Figure 2C). LPS treatment also increased the release of all three pro-inflammatory cytokines (Figure 2). Figure 2 also shows that asprosin exposure, even at a 100 nM concentration, did not stimulate THP-1 macrophages to secrete IL-12β (2D), similarly IL-6 and MCP-1 (Supplementary Figure S1) secretions were not stimulated. These results indicate asprosin regulates the expression and secretion of key inflammatory cytokines.

### 2.2. Asprosin-Induced TNFα Release Is Partly Mediated through the NFκB Pathway

**Figure 3:**
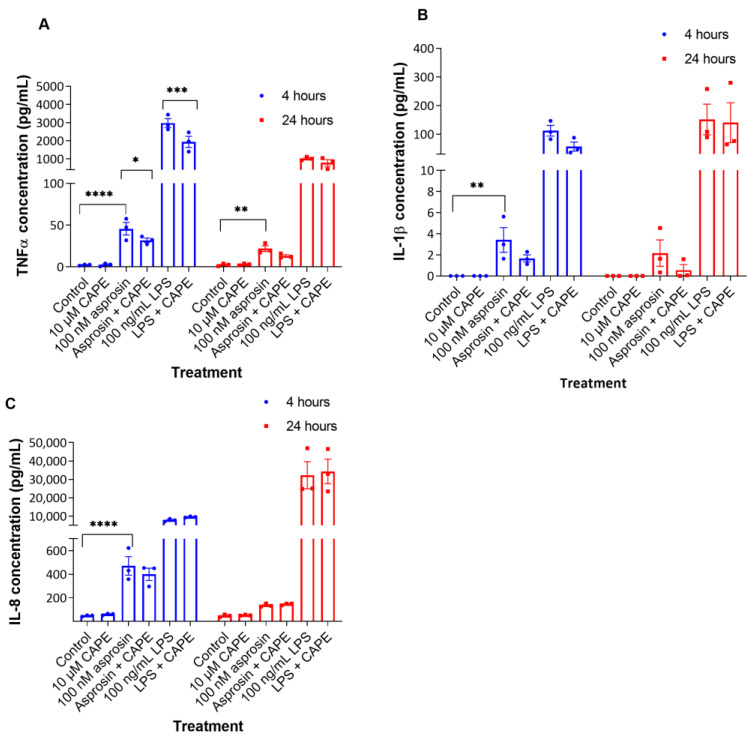
Asprosin-induced release of tumour necrosis factor α (TNFα) is partly mediated through the NFκB pathway. THP-1 macrophages were treated with 10 μM caffeic acid phenethyl ester (CAPE; an inhibitor of NFκB activation), 100 nM asprosin, 10 μM CAPE and 100 nM asprosin, 100 ng/mL lipopolysaccharide (LPS) or both 100 ng/mL LPS and 10 μM CAPE for 4 h and 24 h. Cell supernatants were collected and (**A**) TNFα; (**B**) Interleukin-1β (IL-1β), and (**C**) IL-8 concentrations were measured by ELISA. Data were analysed by two-way ANOVA and Tukey’s multiple comparisons test. Data are presented as mean ± SEM; n = 3; * *p* < 0.05; ** *p* < 0.01; *** *p* < 0.001; **** *p* < 0.0001.

Asprosin has been previously shown to mediate its effects through the NFκB, JNK and AMPK pathways [24,25]. To investigate whether the NFκB pathway was mediating the pro-inflammatory effects of asprosin, CAPE, an established inhibitor of NFκB activation, was used. As previously shown by Vilela et al. (2015) a concentration of 10 µM is not cytotoxic to the THP-1 cells [31], therefore, this CAPE concentration was used in this study. The addition of CAPE did not alter gene expression levels of TNFα compared to 100 nM asprosin without CAPE (Supplementary Figure S2). However, TNFα levels in the supernatant of THP-1 macrophages treated with both 100 nM asprosin and CAPE were significantly lower compared to treatment with 100 nM asprosin alone after 4 h (32 ± 2.68 pg/mL vs. 45.54 ± 7.44 pg/mL, respectively; *p* = 0.033) (Figure 3A). A similar decrease in TNFα levels was noted when cells were treated with LPS and CAPE compared to LPS alone after 4 h (*p* = 0.004) (Figure 3A). These findings confirm that the asprosin-induced TNFα release is mediated partly through the NFκB pathway. Of note, the addition of CAPE did not significantly affect the asprosin-stimulated and LPS-stimulated release of IL-1β (Figure 3B), and IL-8 (Figure 3C) from THP-1 macrophages, suggesting that another pathway is responsible for the release of these cytokines. To elucidate the effect of asprosin on the NFkB pathway, the protein expression of phosphorylated and total NFkB was evaluated by Western blotting. As presented in Supplementary Figure S3, phosphorylated NFkB is upregulated with asprosin (10 nM and 100 nM) stimulation, although statistical significance was not reached (N = 3).

### 2.3. Asprosin Mediates Its Pro-Inflammatory Effects via the TLR4 Pathway

**Figure 4:**
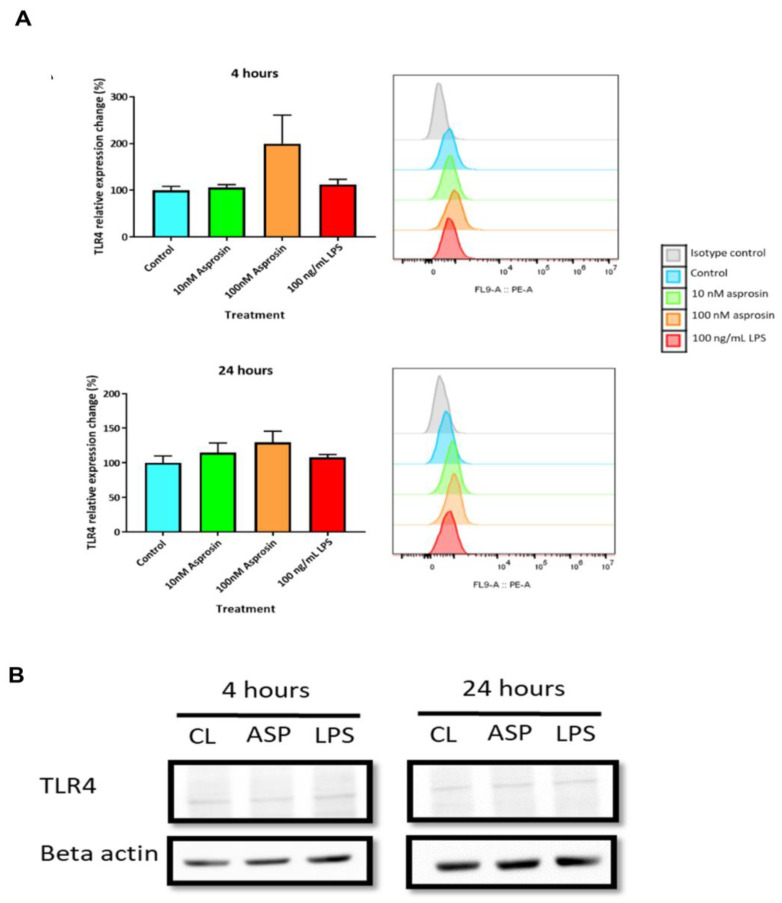
Asprosin did not significantly affect Toll-like receptor 4 (TLR4) surface and intracellular expression. THP-1 macrophages were treated with either 10 nM asprosin, 100 nM asprosin or 100 ng/mL lipopolysaccharide (LPS). Cell surface expression of TLR4 was measured by flow cytometry after 4 and 24 h (**A**). Flow cytometry histograms display fluorescent intensity of the TLR4-PE stain (*x*-axis) and cell count (*y*-axis). Data were compared using one-way ANOVA and Dunnett’s multiple comparisons test (compared to control) (*n* = 3). THP-1-cells (1 × 10^6^ cells/well) were seeded and differentiated into macrophages in 6-well plates. Cells were then treated with either 100 nM asprosin or 100 ng/mL LPS for 4 and 24 h; collected and lysed for intracellular protein expression was measured by Western blotting (**B**). The presented Western data are representative of three independent experiments (*n* = 3). CL: control; ASP: asprosin; LPS: lipopolysaccharide.

In pancreatic beta-cells, Lee and colleagues have demonstrated that the pro-inflammatory effects of asprosin are mediated through the TLR4 and JNK pathway [25]. To investigate this signalling pathway in macrophages, we first studied the effect of asprosin on TLR4 cell surface expression. THP-1 macrophages treated with 100 nM asprosin for 4 h and 24 h showed the greatest change in TLR4 surface expression compared to control cells, but the results were not significant (Figure 4A). Similarly, THP-1 macrophages treated with 10 nM asprosin and LPS did not show any significant change in TLR4 surface expression after 4 and 24 h, compared to unstimulated control cells (Figure 4A). In addition, we measured TLR4 protein levels in THP-1 macrophages treated with 100 nM asprosin and LPS for 4 and 24 h. Figure 4B presents no significant changes in the expression of TLR4 with both treatments at 4 h and 24 h. These data suggest that TLR4 expression is not affected by asprosin or LPS treatment.

**Figure 5:**
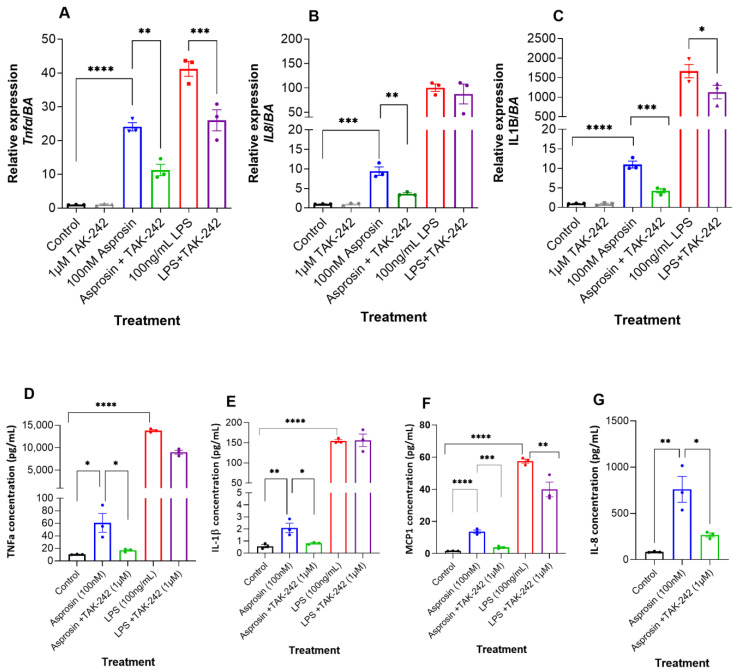
Asprosin-induced inflammation in THP-1 macrophages is inhibited by TAK-242, a Toll-like receptor 4 (TLR4) inhibitor. (**A**) Tumour necrosis factor α (TNFα); (**B**) Interleukin-1β (IL-1β); and (**C**) IL-8 mRNA levels were measured by qRT PCR in THP-1 macrophages treated with 1 μM TAK-242 for 1 h, prior to 4 h stimulation with 100 nM asprosin or 100 ng/mL lipopolysaccharide (LPS). Data are presented as fold-change (mean ± SEM) in transcript. The experiments were repeated in three independent cultures. One-way ANOVA; * *p* < 0.05; ** *p* < 0.01; *** *p* < 0.001; **** *p* < 0.0001. Secreted levels of TNFα (**D**), IL-1β (**E**), MCP-1 (**F**) and IL-8 (**G**) were measured by a human cytokine BioPlex array in cell supernatant of THP-1 macrophages treated with 1μM TAK-242 for 1 h, prior to 4 h stimulation with 100 nM asprosin or 100 ng/mL LPS. The experiments were repeated in three independent cultures. One-way ANOVA; * *p* < 0.05; ** *p* < 0.01; *** *p* < 0.001; **** *p* < 0.0001.

To further elucidate whether, asprosin-induced inflammation affects TLR4 function, we utilized a TLR4 specific inhibitor, TAK-242, which has been shown to inhibit LPS-induced inflammatory pathways in C2C12 myotubes [32]. As demonstrated by Ono and colleagues, we used 1 µM TAK-242 treatment for 1 h on differentiated THP-1 cells prior to stimulation with LPS or asprosin. Data presented in Figure 5 show that TAK-242 significantly inhibits asprosin induced inflammation at both mRNA and secretory levels. Figure 5A shows that TAK-242 significantly inhibited TNFα gene expression in Asprosin+TAK-242 treated samples compared to asprosin-treated samples (*p* = 0.0028, Figure 5A). TNFα expression was also significantly inhibited in LPS+TAK-242 treated samples compared to LPS only treatment (*p* = 0.0006, Figure 5A). IL-8 gene expression was only inhibited in Asprosin+TAK-242 treated samples compared to asprosin-treated samples (*p* = 0.0012, Figure 5B), and were not changed in LPS+TAK-242 treated samples compared to the LPS only treatment (Figure 5B). Figure 5C shows that IL-1β gene expression is significantly inhibited in Asprosin+TAK-242 and LPS+TAK-242 treated samples compared with asprosin and LPS only treatments, respectively (*p* = 0.0004 and *p* = 0.023, respectively; Figure 5C).

Respective cytokine secretion was measured by Bioplex. Figure 5D,E show that TNFα and IL-1β secretion were significantly inhibited with TAK-242 treatment in asprosin treated samples (*p* = 0.023 and *p* = 0.021, respectively). No changes were observed with LPS treatment. Of note, MCP-1 secretion was significantly inhibited by TAK-242 in asprosin treated samples, as well as in LPS treated samples (*p* = 0.0001 and *p* = 0.004, respectively; Figure 5F). IL-8 secretion was only detected with asprosin treatment, and it was significantly inhibited with TAK-242 treatment (*p* = 0.0126, Figure 5G), no IL-8 secretion was detected with LPS treatment. These data suggest that asprosin is mediating its pro-inflammatory effects in THP-1 macrophages through the TLR4 pathway, with TAK-242 treatment significantly inhibiting asprosin-induced cytokine expression and secretion in these cells.

### 2.4. Asprosin Attenuated LPS-Induced Superoxide Release from THP-1 Macrophages

**Figure 6:**
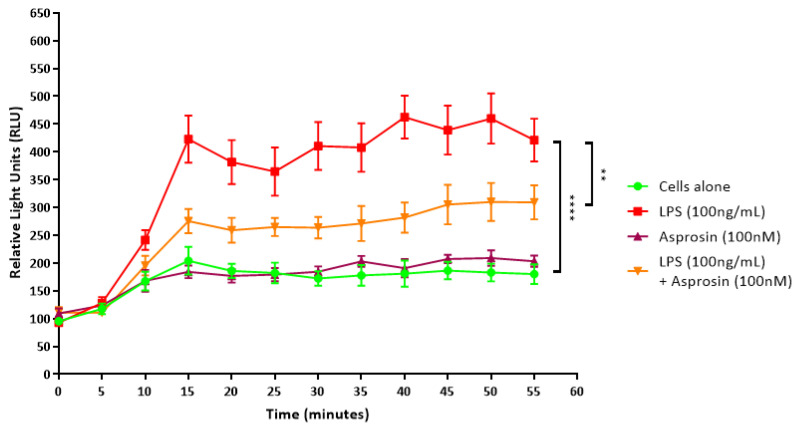
Asprosin attenuates LPS-induced superoxide release. To detect superoxide release, 500,000 THP-1-derived macrophage cells were treated with 20 μM lucigenin and either 100 ng/mL lipopolysaccharide (LPS), 100 nM asprosin or both. Luminescence was measured every 5 min for 55 min. Data were compared using one-way ANOVA and Tukey’s multiple comparisons test (*n* = 3). Data are presented as mean ± SEM; ** *p* < 0.01, **** *p* < 0.0001.

A lucigenin-based assay was also used for the detection of superoxide anions (O2^−^), which react with the lucigenin to produce a luminescent signal that is detected by a luminometer [33]. Within 15 min following LPS treatment, THP-1 macrophages showed a significant increase in superoxide production compared to untreated cells (*p* < 0.0001) (Figure 6). Asprosin treatment alone did not affect the levels of superoxide anions released from cells, but it did significantly lower superoxide release from LPS-treated cells (*p* = 0.0071) (Figure 6). This suggests that asprosin may attenuate LPS-induced oxidative stress.

## 3. Discussion

Chronic low-grade inflammation in obesity is linked to an altered adipokine/cytokine secretion profile by the accumulated excess white adipose tissue, which is dysfunctional and exhibits enhanced immune cell infiltration [6,23]. Asprosin is a novel adipokine which is secreted during fasting conditions and has been found to be increased in the systemic circulation in obesity and other cardio-metabolic diseases [17,23]. The present study demonstrates that asprosin induces a pro-inflammatory response in THP-1 macrophages by significantly promoting the expression and secretion of key pro-inflammatory mediators, namely TNFα, IL-1β, IL-8 and IL-12. Moreover, our present findings further show that treatment with CAPE (an inhibitor of NFκB activation) significantly reduced asprosin-stimulated secretion of TNFα from THP-1 macrophages, whilst treatment with TAK-242 (a TLR4 inhibitor) significantly attenuated asprosin-stimulated TNFα, IL-1β, IL-6 and IL-8 expression and secretion in THP-1 macrophages. Collectively, these novel findings indicate that the TLR4 and NF-kB signalling pathways are implicated in the asprosin-induced pro-inflammatory effects in THP-1 macrophages.

TLR4 constitutes one of the key players of the innate immune system, and is activated in response to an infection, resulting in NF-kB mediated production of pro-inflammatory cytokines, such as TNFα and IL-6 [34,35,36,37,38]. TLR4 is abundantly expressed in macrophages, where LPS-induced autophagy is reported to be regulated through TRIF-dependent and MyD88-independent TLR4 signalling pathways [39]. TAK-242, as a specific TLR4 inhibitor, is shown to bind to Cys747 in the intracellular domain of TLR4 and inhibit the MyD88 and TRIF-dependent pathways [40,41,42]. Previous studies have shown that TAK-242 prevented acute kidney and lung injury in LPS injected animal models [43,44]. More recently, TAK-242 has also been shown to prevent endotoxaemia-induced inflammation in muscle wasting in mice C2C12 myotubes [32]. Moreover, TAK-242 was reported to be well tolerated in a clinical trial for patients with sepsis [45]. Our findings, indicating abolishment of the pro-inflammatory effects of asprosin in THP-1 macrophages treated with TAK-242, add to this existing literature regarding the range of effects that are mediated by the TLR4 signalling pathways.

Notably, in their initial asprosin study, Romere et al. (2016) have suggested that this adipokine could potentially contribute to the onset/exacerbation of diabetes by stimulating hepatic gluconeogenesis through elevating cellular cAMP levels and increasing blood glucose levels [17]. Subsequently, a number of studies have further reported that circulating asprosin levels are significantly increased in individuals with increased body weight (overweightness or obesity), showing positive correlations with body mass index (BMI) and waist circumference, as well as with glycaemic, lipidaemic and pro-inflammatory parameters [24,46,47,48,49,50]. Moreover, the study by Jung et al. (2019) showed that asprosin treatment results in glucose intolerance and insulin resistance in mice, as well as in increased ER stress and circulating levels of pro-inflammatory cytokines, including MCP-1, TNFα and IL-6 [24]. Interestingly, a recent study by Corica et al. (2021) found that asprosin serum levels were significantly lower in children with obesity, and that fasting asprosin decreased with increasing BMI, although it was not significantly affected by insulin resistance [51]. Contrary, Silistre and Hatipogl (2020) found significantly higher serum levels of asprosin in children with obesity compared to normal-weight counterparts, with no gender-related differences [48]. These findings highlight the need for more research focused on elucidating the exact role of asprosin in obesity, T2DM and the chronic pro-inflammatory state that is associated with these conditions.

Furthermore, a recent study by Zou et al. (2022) reported that asprosin inhibits macrophage lipid accumulation in THP-1 derived foam cells by activating ATP binding cassette transporters A1 ABCA1 and ABCG1 via the p38/Elk-1 pathway [52]. This study also found that lentiviral mediated asprosin overexpression decreased the production and secretion of IL-1β and IL-6 in THP-1 macrophage-derived foam cells [52]. In the present study, we found pro-inflammatory effects of asprosin in THP-1 macrophages stimulated with exogenous asprosin, with elevated expression and secretion of TNFα, IL-1β and IL-6. These apparently opposing findings could be partly explained due to the different utilized in vitro experimental settings/models, with presence of lipids in the macrophage foam cells in the study by Zou et al. (2022). Additional research is required to further expand on these findings.

Finally, cardio-protective effects of asprosin have previously been shown, with a study by Wen and colleagues reporting that in vitro asprosin treatment improved the viability of cardiomyoblasts in a dose-dependent manner under hypoxic conditions, whilst it also improved mitochondrial function in H_2_O_2_-treated cells [53]. In line with these data, we also found that asprosin treatment attenuated LPS-induced superoxide release from THP-1 macrophages. Of note, the study by Wen et al. (2020) observed no difference between control cells without H_2_O_2_ treatment and cells treated with asprosin alone, suggesting that asprosin primarily helps restore mitochondrial function under hypoxic conditions [53]. Similarly, asprosin-only treatment did not affect superoxide release in our present study, but when combined with LPS, asprosin significantly reduced superoxide release, suggesting that asprosin may attenuate LPS-induced oxidative stress.

A limitation of our study is that our findings are confined to in vitro experiments on THP-1 macrophages. Further in vivo studies will be required to confirm and expand on our present findings regarding the mediation of asprosin-induced pro-inflammatory effects through the TLR4 and NF-kB pathways. Asprosin is also shown to bind to the GPCR OLFR734/OR4M1 [20,21], thus, further studies should also investigate the possibility of asprosin binding to OR4M1 in THP-1 cells.

## 4. Materials and Methods

### 4.1. Cell Culture and Treatments

THP-1 cells (a human monocytic cell line) were purchased from American Type Culture Collection (#ATCC^®^, TIB-202™). These cells were cultured in Roswell Park Memorial Institute (RPMI)-1640 media (#R0883; Sigma, UK) supplemented with 10% FBS (#F0804; Sigma Aldrich, UK), penicillin (100 μg/mL), and streptomycin (100 units/mL) (#P4333; Sigma Aldrich UK) and 2 mM L-glutmatine ((#G7513; Sigma Aldrich, UK), and were incubated at 37 °C in a humidified environment at 5% CO_2_. To differentiate THP-1 cells into macrophages, 1 × 10^6^ or 5 × 10^5^ cells per well were seeded in 6- and 12-well plates, respectively (Fisher Scientific, UK), and were subsequently treated with 100 nM 1α,25-Dihydroxyvitamin D3 (VD3) (#BML-DM200-0050; Enzo Life Sciences, UK) for 48 h.

THP-1 derived macrophages were then treated with either 1, 10, or 100 nM recombinant human asprosin (#761904; BioLegend, UK) [24,25], 100 ng/mL LPS from Escherichia coli 026:B6 (#15526286; Fisher Scientific, UK) or both 100 ng/mL LPS [29,30] and 100 nM asprosin for 4 and 24 h. After treatment, cells were collected and centrifuged at 300× *g* for 5 min. The supernatants were collected and centrifuged at 2000× *g* for 20 min. Supernatant samples were stored at −20 °C. The cell pellets were stored at −80 °C for RNA and protein extraction.

To investigate the effect of asprosin on NFκB activation, THP-1 derived macrophages were treated for 4 and 24 h with either 10 μM caffeic acid phenethyl ester [31] (CAPE; an inhibitor of NFκB activation) (#2743; Tocris Bioscience, Bristol, UK), 100 nM asprosin, 100 nM asprosin and 10 μM CAPE, 100 ng/mL lipopolysaccharide (LPS) or 100 ng/mL LPS and 10 μM CAPE [31]. Cell pellets and supernatants were then collected and stored in appropriate freezer conditions until analysed.

To investigate the role of TLR4 in asprosin-induced inflammation, THP-1 derived macrophages were treated with 100 nM asprosin or 100 ng/mL LPS with and without a 1 h pre-treatment with 1 μM TAK-242 (R-Ethyl 6-(N-(2-chloro-4-fluorophenyl)sulfamoyl)cyclohex-1-enecarboxylate), a TLR4 signalling inhibitor (#6587; Tocris Bioscience, Bristol, UK). Cell pellets and supernatants were also collected and stored in appropriate freezer conditions until analysed.

### 4.2. SDS-PAGE and Western Blot

Cell pellets were resuspended in RIPA buffer (#20-188; Millipore/MERCK, Welwyn Garden City, UK), sonicated for 10 s, and centrifuged at full speed for 20 min at 4 °C. Protein samples were quantified using the Pierce BCA Protein Assay Kit (#1859078; Thermo ScientificTM, Waltham, MA, USA) and digested (25 µg) with 5× loading buffer (#39000; Thermo ScientificTM, Waltham, MA, USA) at 95 °C for 5 min. Proteins were separated by sodium dodecyl sulphate–polyacrylamide gel electrophoresis and transferred to a PVDF membrane (GE Healthcare, Amersham, UK). The membrane was blocked in blocking buffer 2% bovine serum albumin (BSA) in Tris-buffered saline containing 0.1% Tween-20 for 1 h at room temperature, then incubated in appropriate primary antibodies overnight at 4 °C. Primary antibodies used were: anti-TLR4 derived in mouse (1:500; Biotechne, Abingdon, UK), phosphoNFkB derived in rabbit (1:1000, Cell Signaling Technologies, Danvers, MA, USA), total NFkB derived in rabbit (1/1000, Cell Signaling technologies, Danvers, MA, USA) and HRP conjugated-anti-beta actin derived in mouse (1:1000; Santa Cruz Biotechnology, TA, USA). The membrane was then incubated in appropriate secondary antibodies for 2 h at room temperature. The secondary antibodies used were: anti-mouse-HRP (1:5000; Cell Signaling Technologies, Danvers, MA, USA) and anti-rabbit-HRP (1:5000; Cell Signaling technologies, Danvers, MA, USA). Blots were visualized using Westar Antares Chemiluminescent Substrate (#XLS142; Cyanagen, Bologna, Italy) for Western blotting and the GBOX scanner (Syngene), and the images were analysed using the Image J analysis software (National Institutes of Health, NIH, MD, USA), as previously described [54].

### 4.3. Superoxide Anion Luminometry Assay

THP-1 derived macrophage cells (5 × 10^5^ cells) were transferred into 1.5 mL microcentrifuge tubes and were treated with 20 μM lucigenin (#M8010; Sigma Aldrich, Welwyn Garden City, UK). These cells were either untreated or treated with 100 ng/mL LPS, 100 nM asprosin or both. Zymosan A from Saccharomyces cerevisiae (2 mg/mL) (#Z4250; Sigma Aldirch, Welwyn Garden City, UK) was also added in place of 100 ng/mL LPS. Immediately, the luminescence was measured every 5 min on the GloMax^®^ 20/20 Luminometer (Promega, Southampton, UK) for 55 min, as previously described [33].

### 4.4. Enzyme-Linked Immunosorbent Assays (ELISA)

Human TNFα (#DY210-05), IL-8 (#DY208-05), IL-6 (#DY206-05), 1L-1β/IL-1F2 (#DY201-05), IL-12/p40 (#DY1240-05) and MCP-1 (#DY279-05) DuoSet ELISA kits were purchased from R&D systems (Wiesbaden-Nordenstadt, Germany). Levels of these pro-inflammatory markers in cell culture supernatants were quantified following the corresponding manufacturer’s protocol. All samples were analysed in duplicate, as previously described [54].

### 4.5. Quantitative Gene Expression Analysis by RT-qPCR

Cell pellets were resuspended in 600 μL of Invitrogen™ TRIzol™ reagent (#12044977; Fisher Scientific, UK). Total RNA was isolated from samples using Direct-zolTM RNA MiniPrep (#R2050; Cambridge Biosciences, Cambridge, UK), with a genomic DNA elimination step using DNase I. 500 ng of mRNA was reverse transcribed to cDNA using the Precision nanoScriptTM Reverse Transcription kit (#RT-nanoScript2; Primerdesign Ltd., Eastleigh, UK) according to the manufacturer’s instructions. All primers were ordered from Invitrogen, ThermoFisher (UK). The sequences of primers are presented in Table 1. For one reaction, a master mix was prepared containing 10 μL PrecisionPLUS 2× qPCR mastermix (#PPLUSBLUE-SY-10ML; Primerdesign Ltd., UK), 3 μL RNase/DNase free water, 1 μL sense and anti-sense primers. Expression levels were determined using the LightCycler^®^ 480 (Roche Diagnostics Ltd., West Sussex, UK). Thermal cycling conditions were as follows; denaturation for 10 min at 95 °C, 40 cycles of 15 s at 95 °C and 1 min at 60 °C, followed by melting curve analysis. Beta actin was used as housekeeping gene, and gene expression was calculated using the ∆Ct method described by Livak and Schmittgen [55]. Each sample was analysed in triplicates. Delta Ct (ΔCt) values were obtained by subtracting the Ct values of beta actin from the Ct values of the gene of interest.

### 4.6. Flow Cytometry

Following treatment, cells were collected into 15 mL centrifuge tubes and centrifuged at 300× *g* for 5 min. The supernatant was discarded, and the cell pellets were washed once with 0.1% BSA in PBS. After centrifuging at 300× *g* for 5 min, cells were resuspended in 10% human serum (South American origin) (Gibco, ThermoFisher, UK) in PBS. From each treatment, 2.5 × 10^5^ cells were transferred into 1.5 mL microcentrifuge tubes and 10% human serum/PBS was added for a final volume of 250 μL. Cells were stained with 2.5 μL of antibody. Antibodies used were: CD284 (TLR4) Monoclonal Antibody-PE (HTA125) and Mouse IgG2a kappa Isotype Control (eBM2a)–PE (eBiosciences, ThermoFisher Scientific, UK). Samples were incubated on ice, in the dark for 30 min with the antibodies. Samples were centrifuged at 300× *g* for 5 min, and cells were washed once with 0.1% BSA/PBS. After centrifuging at 300× *g* for 5 min, the pellets were resuspended in 125 μL of 0.1% BSA/PBS. Samples were analysed on the CytoFLEX Flow Cytometer (Beckman Coulter, Indianapolis, IN, USA).

### 4.7. Bio-Plex Multiplex Immunoassay

THP-1 derived macrophages were stimulated with 100 nM asprosin or 100 ng/mL LPS for 4 h with and without a 1 h pre-treatment with 1 μM TAK-242. Cell supernatants were collected, centrifuged at 1500 rpm for 10 min and used to measure the secretory levels of TNFα, IL-1β, IL-8 and MCP-1 by Bioplex-200 using a human cytokine assay (Biorad Technologies, Watford, UK), according to the manufacturer’s instructions.

### 4.8. Statistical Analysis

Data were analysed with the statistical package GraphPad Prism 9 (GraphPad Software Incorporated, La Jolla, CA, USA). One-way ANOVA with post hoc Tukey’s test or Two-way ANOVA tests were used where appropriate, and statistical significance was set at *p*-value < 0.05.

## 5. Conclusions

The findings of the present study offer novel evidence regarding the potential effects of asprosin by demonstrating pro-inflammatory effects of this novel pleiotropic adipokine in THP-1 macrophages, which are mediated, at least in part, by the TLR4 signalling pathway. Indeed, TLR4 inhibition in THP-1 macrophages significantly ameliorates asprosin-induced pro-inflammatory effects, a finding which merits further research attention given the well-established role of chronic inflammation in obesity and obesity-related complications.

## Figures and Tables

**Table 1:** Primers for RT-qPCR.

Gene	Forward	Reverse
TNFα	5′-AGGTTCTCTTCCTCTCACATAC-3′	5′-ATCATGCTTTCAGTGCTCATG-3′
CXCL8 (IL-8)	5′-CAGAGACAGCAGAGCACAC-3′	5′-AGCTTGGAAGTCATGTTTACAC-3
IL-1β	5′-TGGCAATGAGGATGACTTGTTC-3′	5′-CTGTAGTGGTGGTCGGAGATT-3′
IL-12β	5′-CTCACCCCCACCTCTCTAAAA-3′	5′-TGTCCTTAGCCATAACTACTTGTC-3′
β-actin	5′-CTGGAACGGTGAAGGTGACA-3′	5′-AAGGGACTTCCTGTAACAATGCA-3

## Data Availability

Data are contained within the paper and its Supplementary Material.

## Supplementary Materials

The following supporting information can be downloaded at: https://www.mdpi.com/article/10.3390/ijms24010227/s1.
