# Supplementary material for: Asprosin Exerts Pro-Inflammatory Effects in THP-1 Macrophages Mediated via the Toll-like Receptor 4 (TLR4) Pathway
Source: Int J Mol Sci. 2022 Dec 23;24(1):227. doi: 10.3390/ijms24010227 (PMC9820073; doi:10.3390/ijms24010227)
Supplement: Supplementary file 1 [file ijms-24-00227-s001.zip › ijms-2089815-supplementary.pdf]

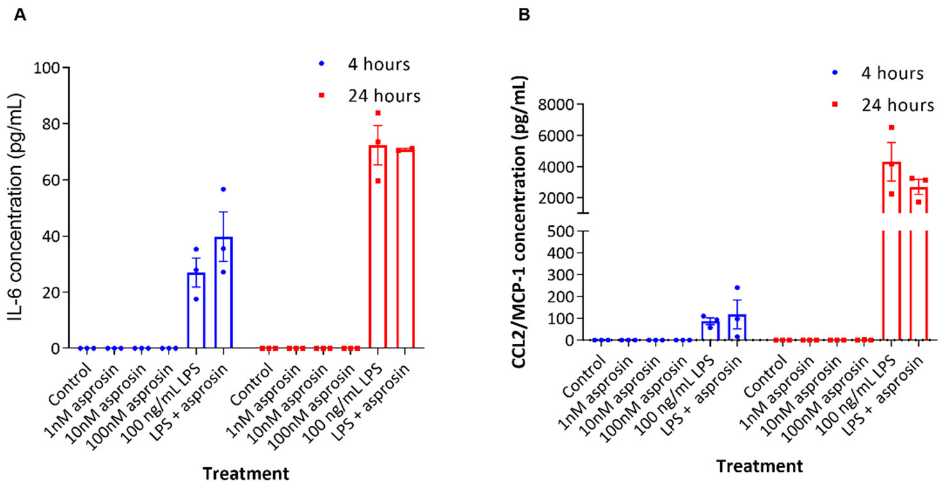

Figure S1. Secretion of interleukin-6 (IL6) and monocyte chemoattractant protein-1 (MCP-1) in response to Asprosin and LPS treatment. THP-1 macrophages were treated with increasing concentrations of asprosin (1 nM, 10 nM, and 100 nM), 100 ng/mL lipopolysaccharide (LPS) or both 100 ng/mL and 100 nM asprosin for 4 hours and 24 hours. Cell supernatants were collected and (A) IL6 and, (B) MCP-1 concentrations were measured by ELISA. Data were analysed by two-way ANOVA and Tukey's multiple comparisons test (compared to respective controls). Data are presented as mean±SEM; n=3.

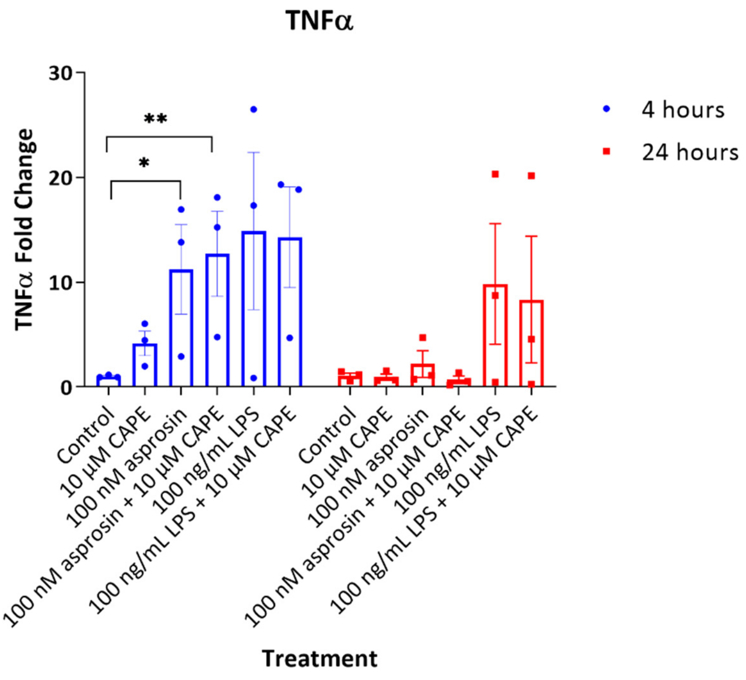

Figure S2. Tumour necrosis factor alpha (TNF*α*) gene expression with caffeic acid phenethyl ester (CAPE) treatment. THP-1 macrophages were treated with 10 µM CAPE) an inhibitor of NFκB activation), 100 nM asprosin, 10 µM CAPE and 100 nM asprosin, 100 ng/mL lipopolysaccharide (LPS) or both 100 ng/mL LPS and 10 µM CAPE for 4 hours and 24 hours. Gene expression of TNF*α* was measured by RT-qPCR; Data were analysed by two-way ANOVA and Tukey's multiple comparisons test. Data are presented as mean±SEM; n=3; *p<0.05; **p<0.01.

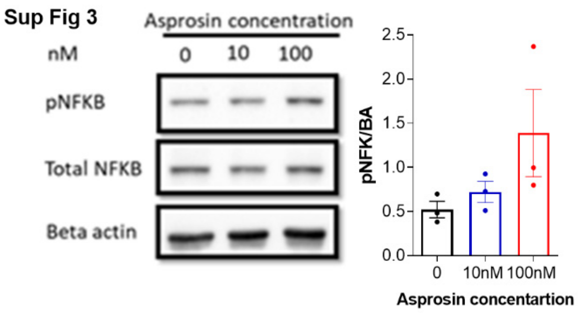

Figure S3. Phosphorylation of NFκB in THP-1 macrophages following asprosin treatment. Phosphorylated and total NFκB were determined by western blot analysis in THP-1 macrophages treated with 10 nM or 100 nM asprosin for 15 minutes. Beta actin was used a loading control and relative intensity was determined by densitometry. The experiments were repeated in three independent cultures and data were analysed using one-way ANOVA and Tukey's multiple comparisons test.
